# Improvement of Ocean State Estimation by Assimilating Mapped Argo Drift Data

**DOI:** 10.1155/2014/975618

**Published:** 2014-07-15

**Authors:** Shuhei Masuda, Nozomi Sugiura, Satoshi Osafune, Toshimasa Doi

**Affiliations:** Research and Development Center for Global Change, Japan Agency for Marine-Earth Science and Technology (JAMSTEC), Natsushima 2-15, Yokosuka, Kanagawa 237-0061, Japan

## Abstract

We investigated the impact of assimilating a mapped dataset of subsurface ocean currents into an ocean state estimation. We carried out two global ocean state estimations from 2000 to 2007 using the K7 four-dimensional variational data synthesis system, one of which included an additional map of climatological geostrophic currents estimated from the global set of Argo floats. We assessed the representativeness of the volume transport in the two exercises. The assimilation of Argo ocean current data at only one level, 1000 dbar depth, had subtle impacts on the estimated volume transports, which were strongest in the subtropical North Pacific. The corrections at 10^°^N, where the impact was most notable, arose through the nearly complete offset of wind stress curl by the data synthesis system in conjunction with the first mode baroclinic Rossby wave adjustment. Our results imply that subsurface current data can be effective for improving the estimation of global oceanic circulation by a data synthesis.

## 1. Introduction

The synthesis of observational data and model results to yield an estimate of the ocean state, particularly through the use of a smoothing method, is a promising approach to better understanding of climate change [1, 2]. Developments in computer science have made long-term state estimation of the global ocean a tractable problem [3–5].

Observations are crucial for determining the quality of ocean state estimations. The introduction of Argo profiling floats in the early 2000s significantly enhanced understanding of ocean climate variations [6]. The resulting global profiles of water temperature and salinity have become indispensable inputs for ocean state estimations.

Current data and surface pressure distributions are promising for input data. One recent study investigated the impact of using bottom pressure anomalies obtained from remote sensing data, in addition to mean sea surface heights, as an index of surface currents [7]. These have had various degrees of success in improving state estimations.

Argo floats provide information on their drift speed at their parking depth as well as at the sea surface [8]. Katsumata and Yoshinari [9] developed a scheme to derive from this information a map of average subsurface currents at 1000 dbar depth over the period 2000–2010.

In this study, we examined the impact on an ocean state estimation of assimilating a dataset of subsurface ocean currents derived from Argo floats. Our focus in this paper is on how this assimilation affected the representativeness of volume transport in an ocean state estimation in conjunction with basin-scale circulation.

## 2. Data and Methods

### 2.1. Mapped Argo Drift Data

**Figure 1:**
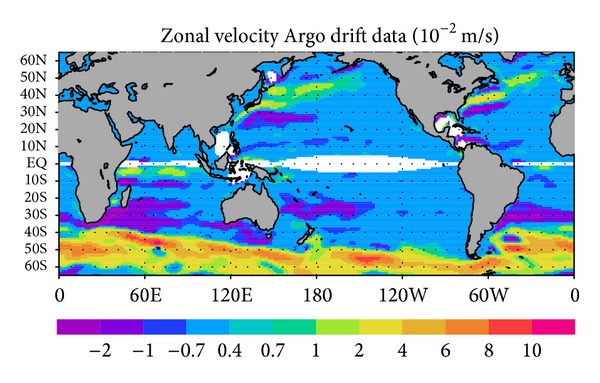
Annual mean zonal velocity at 1000 dbar (eastward is positive) derived from Argo drift data during 2000–2010 [9].

Katsumata and Yoshinari [9] derived the global map of geostrophic velocity “G-YoMaHa” from drift data of Argo floats at their parking level (http://www.jamstec.go.jp/ARGO/argo_web/G-YoMaHa/index_e.html).  Figure 1 shows the resulting velocity field at a depth of 1000 dbar. Because data for global oceanic velocities are sparse for subsurface levels, the Argo data provide important observational information for subsurface velocity fields, although it should be noted that the G-YoMaHa dataset includes typical errors of 3.0 × 10^−2^ m s^−1^.

### 2.2. Ocean Data Synthesis System

The data synthesis system was developed as a part of the K7 consortium between the Japan Agency for Marine-Earth Science and Technology (JAMSTEC) and Kyoto University. The ocean general circulation model (OGCM) applied to this system was based on version 3 of the Geophysical Fluid Dynamics Laboratory (GFDL) Modular Ocean Model (MOM) [10]. The horizontal resolution is 1° in both latitude and longitude, and the global ocean basin has 46 vertical levels. The data synthesis method is based on a four-dimensional variational (4D-Var) strong constraint approach [11], with adjoint code from the global OGCM. This system seeks the optimal solution (the best time trajectory of the model results) by synthesizing the OGCM results and observational data within the framework of the model formalism and thus provides a dynamically self-consistent dataset [12, 13].

We made two ocean state estimations, the first one using the G-YoMaHa subsurface ocean velocity field at 1000 dbar from Argo drift data (Figure 1) as input (the GY case) and the second one, not using G-YoMaHa, as a control case (CT). The weight of assimilation was determined on the basis of information on uncertainty provided by Katsumata and Yoshinari [9]. The assimilation window was the 8-year period from 2000 to 2007. The other numerical implementations and experimental settings, inclusive of input data, were the same as those reported by Kouketsu et al. [13].

## 3. Results

**Figure 2:**
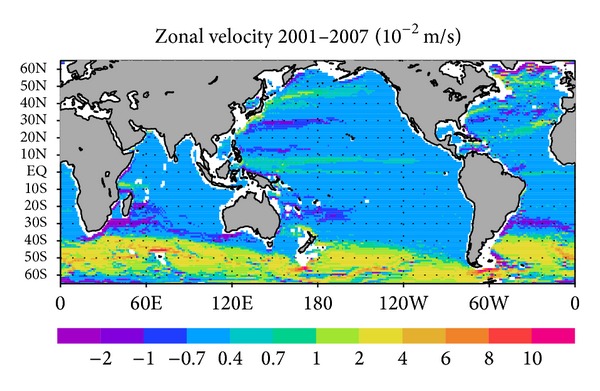
Estimated mean zonal velocity during 2001–2007 at a depth of 1000 m in the GY case.

The zonal velocity field in the GY case at a depth of 1000 m (approximately 1000 dbar; Figure 2) is in basic agreement with that of G-YoMaHa (Figure 1). This is not a trivial result in a strong constraint 4D-Var approach, where the model is assumed to be perfect. The two velocity fields are consistent in their overall pattern and magnitudes. There are some discrepancies in the open ocean region, particularly in the Southern Ocean, but the GY field is generally acceptable as a basic ocean state, considering that the uncertainties are on the order of 1.0 × 10^−2^ m s^−1^ and that these include the intrinsic error arising from the sparseness of the data [9].

**Figure 3:**
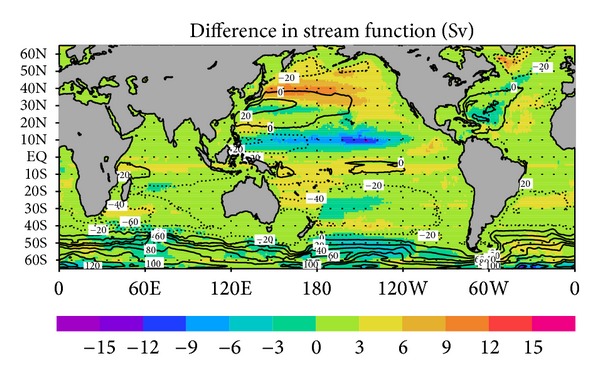
Difference in the mean barotropic volume transport function (in sverdrups) during 2001–2007 between the GY and CT cases (color) and mean value of barotropic volume transport function for the CT case (contours).

Figure 3 shows the difference in barotropic volume transport function between the GY and CT cases. The volume transport is calculated by integrating the depth-integrated meridional velocity. There are particularly large differences in regions in the North Pacific centered on 10°N and 40°N, which are relevant to intensification of the North Equatorial Current and reduction of the Kuroshio extension, as well as in some local regions around the Drake Passage and in the northern North Atlantic.

These results show that the assimilation of G-YoMaHa data has a subtle impact on the modeled gyre circulation of the North Pacific. The locations affected are likely related to the measurement depth (in this case 1000 dbar), although further investigations are required to clarify this point.

**Figure 4:**
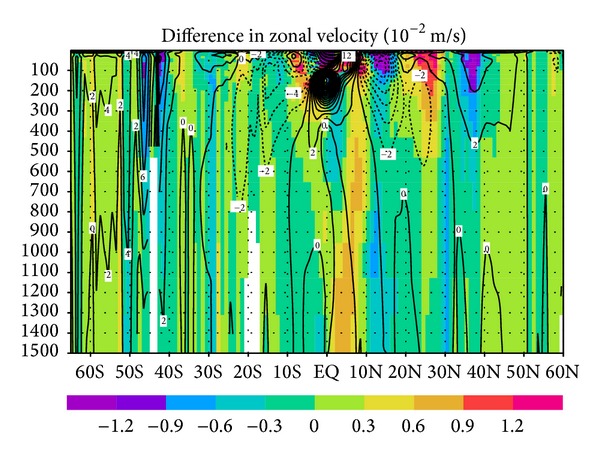
Difference in mean zonal velocity (10^−2^ m s^−1^) during 2001–2007 between GY and CT cases (color) and mean value for the CT case (contours) in a 1500 m vertical cross section along longitude 180°.

**Figure 5:**
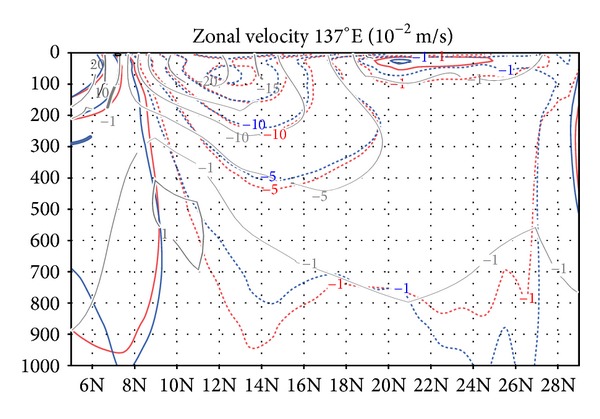
Mean zonal velocity (10^−2^ m s^−1^) during 2001–2007 for the GY (red curves) and CT (blue) cases in a 1000 m vertical cross section along longitude 137°E. Gray curves show the climatological geostrophic velocity field calculated by Zhai and Hu [14].

Figure 4 shows the difference between the GY and CT cases in zonal velocity across longitude 180° in the upper 1500 m of the water column. The corrections in the velocity field are greatest (more than 1.0 × 10^−2^ m s^−1^) in the upper 400 m between 10°N and 40°N in the North Pacific. This change is largely consistent with the results for the stream function in Figure 3. We here evaluate the velocity field in the GY case by comparing it to a vertical section of annual mean zonal geostrophic velocity at 137°E that was calculated by Zhai and Hu [14] from water temperature and salinity observations by the Japan Meteorological Agency (JMA). Our experiment does not directly synthesize this velocity field, although we include a fraction of the JMA observations as assimilated elements. In this vertical section, incorporation of the G-YoMaHa data generally deepens the major current systems in the upper 500 m depth range by dozens of meters (red and blue curves in Figure 5). This tendency improves our model's consistency with observations (gray curves), particularly in the region between 14°N and 26°N, and hence it may improve the state estimation.

**Figure 6:**
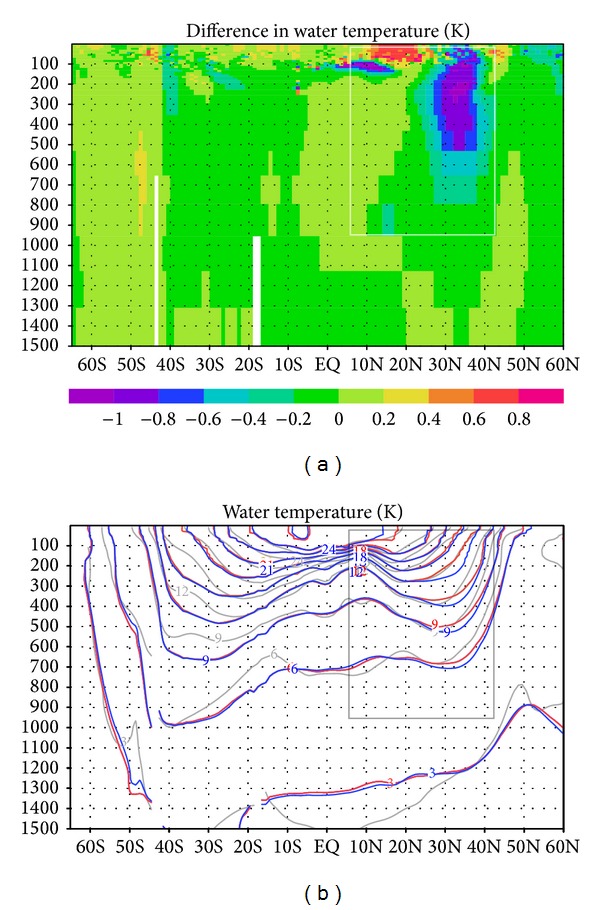
(a) Difference in mean water temperature (K) during 2001–2007 between GY and CT cases in a 1500 m vertical cross section along longitude 180°. (b) Mean water temperature for GY (red curves) and CT (blue) cases in a 1500 m vertical cross section along longitude 180°. Gray curves are mean values from the MOAA GPV observation dataset [15].

The water temperature field is also influenced by the assimilation of the subsurface current data, in particular in the upper North Pacific (the white outline in Figure 6(a)). For instance, in the upper 600 m of the water column between 30°N and 40°N, the subsurface water temperature in the GY case is 0.6–1.0 K cooler than in the CT case owing to the changes in the circulation. We validated our synthesis results against water temperature and salinity observations from the dataset Grid Point Value of the Monthly Objective Analysis using Argo data (MOAA GPV) [15]. MOAA GPV is a comprehensive dataset based on subsurface observations, which was not directly used in our data synthesis.  Figure 6(b) shows that water temperature in the GY case (red curves) is closer to MOAA GPV (gray curve) than in the CT case (blue curve) and is by and large consistent with MOAA GPV in the region where the influence of assimilation is greatest (gray outline).

**Figure 7:**
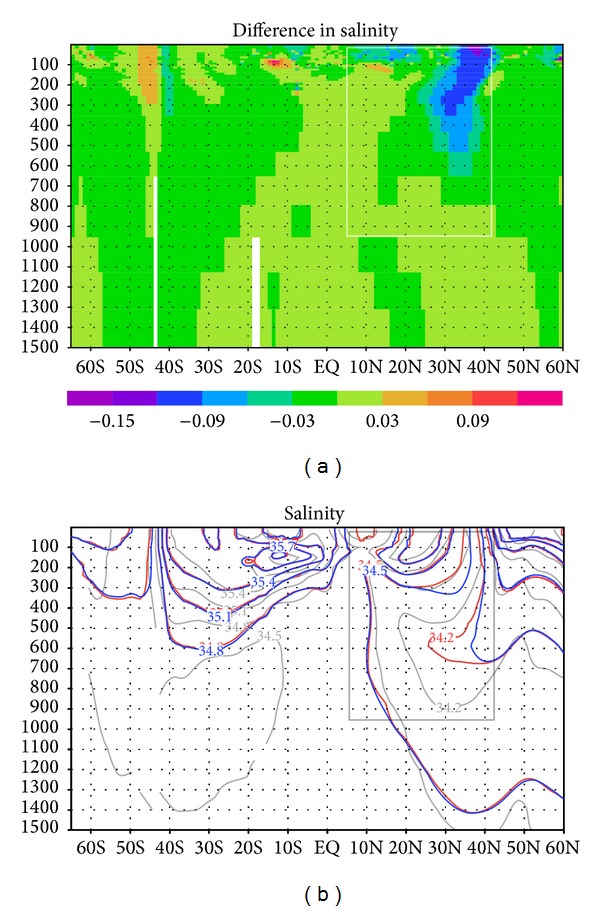
(a) Difference in salinity during 2001–2007 between GY and CT cases in a 1500 m vertical cross section along longitude 180°. (b) Mean salinity for GY (red curves) and CT (blue) cases in a 1500 m vertical cross section along longitude 180°. Gray curves are mean values from the MOAA GPV observation dataset [15].

The salinity field displays the same tendency as the temperature field (Figure 7). The North Pacific Intermediate Water, characterized by a salinity minimum of 34.2, is better reproduced in the GY case. These results again imply that assimilation of mapped subsurface current data can improve the quality of ocean state estimations, not only in velocity but also in other physical parameters.

**Figure 8:**
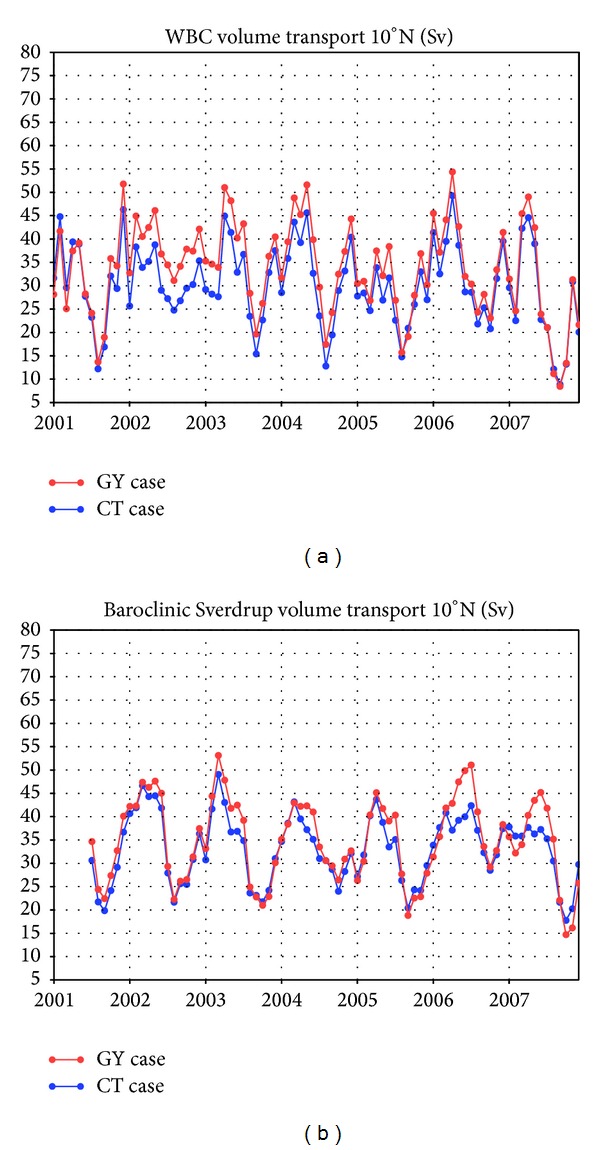
Time series of (a) the estimated southward volume transport (in sverdrups) of the western boundary current across 10°N and (b) the northward “baroclinic” Sverdrup volume transport at 10°N [16].

Figure 8 demonstrates how our data synthesis system generated these differences in the North Pacific region.  Figure 8(a) shows that the time series of volume transport by the southward western boundary current across 10°N is similar in both the GY and CT cases, although the mean value in the GY case is 2.0 Sv larger than the mean in the CT case.  Figure 8(b) shows the time series of the northward “baroclinic” Sverdrup volume transport across 10°N [16] calculated using only the estimated wind stress fields in the GY and CT cases under the assumption of a constant phase (group) velocity of baroclinic Rossby waves of 2.9 × 10^−1^ m s^−1^ at the first baroclinic Rossby radius of deformation of 120 × 10^3^ m [17]. Note that this basin-scale adjustment is not expected to be complete before June 2001 under the assumption of such a Rossby wave starting from the eastern boundary at the January 2000 start time of this experiment. The variations are in basic agreement with those for the western boundary current (Figure 8(a)). The difference in the mean value of baroclinic transport between the GY and the CT cases is 1.8 Sv, which can account for 90% of the difference between the two cases in the western boundary current. This shows that the assimilation of subsurface current data at one specific level effectively offsets the wind stress curl throughout this system.

These two time series also show discrepancies in their interannual variations. For instance, the influence of the assimilation on the western boundary current volume transport is greatest in 2002 (Figure 8(a)), whereas its influence on the baroclinic transport is at its maximum during 2006-2007 (Figure 8(b)). The discrepancy may stem from the assumptions underlying the baroclinic transport calculations and also from the definition of the assimilation window and the control variables of the synthesis system. A more detailed discussion of these factors, however, is beyond the scope of this paper.

## 4. Conclusion

We assessed the impact of adding subsurface velocity data to an ocean state estimation by comparing estimates made with and without the G-YoMaHa dataset. The impact was notable in the North Pacific region, for instance, as an intensification of the gyre circulation at 10°N, achieved through the nearly complete correction of wind stress curl in conjunction with the baroclinic oceanic adjustment. Although our results may depend on specifics of the model platform such as its formalism, physical schemes, or resolution, the incorporation of subsurface current data even at one specific level can improve ocean state estimations at the basin scale. Further work along these lines may enable us to identify the key levels to be monitored and the optimal requirements for measurement instruments to be deployed in the future.

## References

[1] Wunsch C (1996). *The Ocean Circulation Inverse Problem*.

[2] Wunsch C, Heimbach P, Siedler G, Griffies S, Gould J, Church J (2013). Dynamically and kinematically consistent global ocean circulation and ice state estimates. *Ocean Circulation and Climate: A 21st Century Perspective*.

[3] Stammer D, Wunsch C, Giering R (2002). Global ocean circulation during 1992–1997, estimated from ocean observations and a general circulation model. *Journal of Geophysical Research C: Oceans*.

[4] Köhl A, Stammer D (2008). Variability of the meridional overturning in the North Atlantic from the 50 years GECCO state estimation. *Journal of Physical Oceanography*.

[5] Masuda S, Awaji T, Toyoda T, Shikama Y, Ishikawa Y (2009). Temporal evolution of the equatorial thermocline associated with the 1991–2006 ENSO. *Journal of Geophysical Research*.

[6] Koblinsky CJ, Smith NR, Argo Science Team (2001). Argo: the global array of profiling floats, in observing the oceans in the 21st century. *GODAE Project Office*.

[7] Köhl A, Siegismund F, Stammer D (2012). Impact of assimilating bottom pressure anomalies from GRACE on ocean circulation estimates. *Journal of Geophysical Research*.

[8] Lebedev KV, Yoshinari H, Maximenko NA, Hacker PW (2007). YoMaHa07: velocity data assessed from trajectories of Argo floats at parking level and at the sea surface. *IPRC Technical Note*.

[9] Katsumata K, Yoshinari H (2010). Uncertainties in global mapping of Argo drift data at the parking level. *Journal of Oceanography*.

[10] Pacanowski RC, Griffies SM (1999). *The MOM 3 Manual*.

[11] Sasaki Y (1970). Some basic formalisms in numerical variational analysis. *Monthly Weather Review*.

[12] Masuda S, Awaji T, Sugiura N (2010). Simulated rapid warming of abyssal North Pacific waters. *Science*.

[13] Kouketsu S, Doi T, Kawano T (2011). Deep ocean heat content changes estimated from observation and reanalysis product and their influence on sea level change. *Journal of Geophysical Research*.

[14] Zhai F, Hu D (2013). Revisit the interannual variability of the North Equatorial current transport with ECMWF ORA-S3. *Journal of Geophysical Research: Oceans*.

[15] Hosoda S, Ohira T, Nakamura T (2008). A monthly mean dataset of global oceanic temperature and salinity derived from Argo float observations. *JAMSTEC Report of Research and Development*.

[16] Akitomo K, Ooi M, Awaji T, Kutsuwada K (1996). Interannual variability of the Kuroshio transport in response to the wind stress field over the North Pacific: its relation to the path variation south of Japan. *Journal of Geophysical Research C*.

[17] Chelton DB, Deszoeke RA, Schlax MG, El Naggar K, Siwertz N (1998). Geographical variability of the first baroclinic Rossby radius of deformation. *Journal of Physical Oceanography*.

